# Peripheral Neuropathy under Oncologic Therapies: A Literature Review on Pathogenetic Mechanisms

**DOI:** 10.3390/ijms22041980

**Published:** 2021-02-17

**Authors:** Mariarita Laforgia, Carmelo Laface, Concetta Calabrò, Simona Ferraiuolo, Valentina Ungaro, Domenico Tricarico, Cosmo Damiano Gadaleta, Patrizia Nardulli, Girolamo Ranieri

**Affiliations:** 1 Pharmacy Unit, IRCCS Istituto Tumori “Giovanni Paolo II”, Viale Orazio Flacco 65, 70124 Bari, Italy; m.laforgia@oncologico.bari.it (M.L.); concetta.calabro@oncologico.bari.it (C.C.); s.ferraiuolo@oncologico.bari.it (S.F.); valentina_ungaro@hotmail.com (V.U.); p.nardulli@oncologico.bari.it (P.N.); 2 Interventional and Medical Oncology Unit, IRCCS Istituto Tumori “Giovanni Paolo II”, Viale Orazio Flacco65, 70124 Bari, Italy; carmelo.laface@gmail.com (C.L.); c.gadaleta@oncologico.bari.it (C.D.G.); 3 Department of Biomedical Sciences and Human Oncology, University of Bari ‘Aldo Moro’, 70121 Bari, Italy; 4 Section of Pharmacology, Department of Pharmacy-Pharmaceutical Sciences, University of Bari, Via Orabona 4, 70125 Bari, Italy; domenico.tricarico@uniba.it

**Keywords:** neurotoxicity, anti-cancer drugs, pharmacology, chemotherapy-induced peripheral neuropathy, neurodegeneration, mechanism

## Abstract

Peripheral neurologic complications are frequent adverse events during oncologic treatments and often lead to dose reduction, administration delays with time elongation of the therapeutic plan and, not least, worsening of patients’ quality of life. Experience skills are required to recognize symptoms and clinical evidences and the collaboration between different health professionals, in particular oncologists and hospital pharmacists, grants a correct management of this undesirable occurrence. Some classes of drugs (platinates, vinca alkaloids, taxanes) typically develop this kind of side effect, but the genesis of chemotherapy-induced peripheral neuropathy is not linked to a single mechanism. This paper aims from one side at summarizing and explaining all the scattering mechanisms of chemotherapy-induced peripheral neuropathy through a detailed literature revision, on the other side at finding new approaches to possible treatments, in order to facilitate the collaboration between oncologists, hematologists and hospital pharmacists.

## 1. Introduction

Peripheral Nervous System is the *trait d’union* between brain and spinal cord (Central Nervous System) and the rest of the body, granting the passage and transmission of information to transform in actions. Back to CNS, peripheral nerves send sensory information deriving from the external world.

Peripheral neuropathy (PN) is a more or less serious damage to the peripheral nerves (sensory, motor and autonomic nerves), affecting the Aβ (myelinated fibers involved in touch and vibration sense), Aδ (thinly myelinated fibers involved in cold perception and thermal pain), and C-fiber (unmyelinated fibers involved in warm temperature perception and thermal pain) functions [1]. It is often accompanied by weakness, numbness and pain, frequently in hands and feet, described by patients as stabbing, burning or tingling, often spreading upward to arms and legs [2]. Other symptoms of PN are extreme sensitivity to touch, pain during normal activities [3], lack of coordination and falling, muscle weakness, paralysis if motor nerves are affected [4,5], while heat intolerance, excessive or lack of sweating, bowel, bladder or digestive problems, changes in blood pressure [6], dizziness or lightheadedness if autonomic nerves are bit [7]. PN can affect a single nerve (mononeuropathy), two or more nerves in different areas (multiple mononeuropathy) or many nerves (polyneuropathy) [8].

Exposure to toxins is one of the principal causes of PN. In particular, for some classes of anticancer drugs, PN represents the most frequent cause of therapy interruption, dose reduction, delay and variation of the standard timing of administration, till leading to not optimal clinical outcomes.

The global (acute and chronic) prevalence of Chemotherapy-Induced PN (CIPN), in all its clinical manifestations (sensorial, motor and autonomic) is agent-dependent and, according to 2014 data deriving from a systematic review and meta-analysis by Seretny [9], varies from 68.1% in the first month after chemotherapy to 60% at three months and 30% at six months and more. The highest values have been recorded for platinum-based drugs (70–100%) [5,10], taxanes (up to 76%) [11], thalidomide and its analogues (20–60%) [12,13] and ixabepilone (66%) [14]. More specifically, Sisignano asserted that the neuropathic pain related to chemotherapy treatments affected up to 80% of patients [15]. These data are expression of a huge clinical impact of CIPN on oncologic patients and have grown up in a few years.

Sensorial and motor alterations represent the most frequent forms of CIPN symptoms and are associated to most drugs (taxanes, platinum salts), involving both large and small fibers, while autonomic manifestations are less frequent and are related predominantly to vinka alkaloyds (small fibers damage) [16,17,18].

Platinum-based antineoplastic drugs (oxaliplatin and cisplatin), taxanes (paclitaxel, docetaxel), epothilones (ixabepilone), immunomodulatory drugs (thalidomide), vinca alkaloids (vincristine and vinblastine), proteasome inhibitors (bortezomib) and tyrosine kinase inhibitors (sorafenib) are, in approximately decreasing neurotoxic impact order, the classes of antineoplastics that more frequently are associated to CIPN development [19]. Nevertheless, also innovative therapeutic approaches, such as Chimeric Antigen Receptor-T (car-T) cell therapy, in particular tisagenlecleucel used for the treatment of relapsed/refractory diffuse large B-cell lymphoma, has demonstrated to induce a certain grading of neurological toxicity [20].

Different risk factors must be considered to forecast or prevent the neuropathic side effects associated to the chosen anticancer therapy: smoking, advanced age, increased body mass index, uncontrolled diabetes, alcohol abuse vitamin B deficiencies, pre-existing infections (Lyme disease, shingles, Epstein-Barr virus, hepatitis B/C, HIV), autoimmune diseases, (rheumatoid arthritis, lupus), kidney, liver or thyroid disorders, family history of neuropathy [8,9,21,22,23]. Cold allodynia and cold hyperalgesia during chemotherapy has also been identified as a risk factor for persistent CIPN symptoms [9]. Also single nucleotide polymorphisms (SNPs) [8], evidenced during genome-wide association studies (GWAS) have demonstrated to bring a higher risk of CIPN, in particular referring to voltage-gated sodium channels, Schwann cell function–related proteins, receptors for cell surface collagen, receptors involved in neuronal apoptosis, neuronal crest cell development and even an enzyme involved in pyruvate metabolism [9].

Severity of CIPN symptoms varies also as a function of the drug administered and its schedule of treatment and cumulative dose [19], i.e., platinum salts, specifically oxaliplatin, induce immediate acute severe sensory disturbances, while the most frequent taxane-induced neurotoxicity (paclitaxel more than docetaxel) generates longlasting but less severe symptoms [24,25]. No standard management of symptoms is recognized at the moment, though a single pharmacological treatment has been postulated for all CIPN, by oxaliplatin, cisplatin, vincristine and paclitaxel, because of similar overlapping mechanisms of mitotoxicity [26,27], involving the inhibition of TRPV1 [28] or NMDAR [29] activation with additional anti-inflammatory properties, perhaps by inhibiting p38-MAPK [30].

This review aims at summarizing and explaining all the scattering mechanisms of CIPN onset through a detailed literature revision. The knowledge of toxicity profiles and the pathogenetic mechanisms of CIPN onset of old and new drugs in a multidisciplinary dialogue represents a due for all professionals during patients’ care path. Experience skills are required to recognize symptoms and clinical evidences and the collaboration between different health professionals, in particular oncologists and hospital pharmacists, grants a correct management of this undesirable occurrence. This research has been the starting point for deepening our knowledge on the topic as professionals within a cancer institute and beginning a constant collaboration for the success of the therapies.

## 2. Materials and Methods

A systematic literature research about chemotherapy-induced peripheral neuropathy has been collected with special referring to the reviews on this topic. The keywords used during research were *peripheral neuropathy, neurotoxicity, mechanism of neuropathy onset, neuropathic pain* associated to taxanes, platinum salts, vinca alkaloyds, bortezomib and TKIs. During the data collecting, Thalidomide’s and Ephotilones’data have been found interesting and also inserted in the manuscript, to shed light on all possible mechanisms of CIPN onset.

## 3. Results

Our research has covered a period of about ten years, since 2010 and all the publications have been chosen in order to gain completeness of information on mechanisms of onset, clinical data (prevalence, data emerging from clinical trials), possible prevention or treatment with or without therapeutic agents, genetic studies about the possible risk factors predisposing to CIPN. The information about thalidomide in monotherapy are older than the others, involving papers published before 2010, nevertheless more recent reports have been inserted about the combination therapy of thalidomide and bortezomib in multiple myeloma. Within our hospital and our daily activity, this research has brought us to give to oncologists and hematologists a sort of booklet to help them to prescribe the right drug, timing of administration and dosage, based on the patient’s pre-existing comorbidities. On the other hand, hospital pharmacists could enrich their counseling towards patients reporting neuropathic symptoms, suggesting possible alternative remedies or sharing with the physician a possible chrono-modulation of the drug administration.

## 4. Discussion

The study has been conceived as a summit of the knowledge about CIPN induced by the most common neurotoxic anticancer drugs, in order to give them a more right clinical collocation in therapeutic schedules of oncologic patients, which often are affected by different comorbidities susceptible to alter or modify clinical outcomes in terms of toxicity, delay or suspension of their treatments. With respect to already published papers on this topic, our study combines more aspects of this important problematic for the patients and the physicians facing neuropathic symptoms: mechanisms of onset, risk factors, pharmacologic or not pharmacologic treatments, studies upon genetic patterns, possible variation in dose and timing of administration. This is a review of literature, but it has given us the possibility to start collecting a growing casuistry, among all patients in our cancer institute. In the following part of the discussion, we report the principal pharmacological and toxicological characteristics of PN-induced anticancer drugs, summarized in Table 1.

### 4.1. Taxanes

Chemotherapy based on taxane drugs (paclitaxel, docetaxel, cabazitaxel) is largely prescribed, often with curative intent, in breast, ovarian, lung and prostate cancer [31]. Taxane neuropathy affects 80% to 97% of patients and among then, 27% complaints neuropathic pain [32,33]. A retrospective study [134] indicates that about 10% of patients under taxane treatment, undergo a dose reduction or delays in administration timing because of TIPN (taxane-induced peripheral neuropathy) symptoms. Other studies have estimated that 25% to 60% of all patients will experience lasting neuropathic symptoms months to years after the completion of chemotherapy [34,35] associated with anxiety, sleep disorders and depression [135].

Taxanes’ pharmacodynamics involves microtubule-stabilizing mechanisms, prevent thanks to their high affinity with the interior portion of the b-tubulin subunit that block mitosis and tumour cell proliferation. Different timing and schedules of treatment are known and are applicable to different patient’s performance status and hematologic pattern, besides referring to neoadjuvant/adjuvant strategy or metastatic disease. Paclitaxel is more neurotoxic than docetaxel and its newer formulation, albumin-bound paclitaxel (Nab-paclitaxel), cannot succed in reducing CIPN incidence though it has reduced overall toxicity [36]. TIPN is usually a sensory dominant neuropathy, mostly affecting small diameter fibers, while motor and autonomic involvement is less frequent [37,38].

Tingling, itching, burning, spontaneous pain, heightened sensitivity to touch (positive sensory symptoms) or loss of touch and pain and loss of hot/cold sensations (negative sensory symptoms) can be both experienced in the extremities (hands and feet) and generally worsen with increasing dose and treatment duration [32].In some patients, symptoms continue up to 1–3 years after therapy and sometimes are lifelong lasting [39].

The negative sensory profile is subsequent to the loss of intraepidermal nerve fiber density and demyelination [40,41,42,136], besides a degeneration of distal nerve segments (Wallerian degeneration) and an axonal membrane remodelling [137]. Animal models consented to prove this effect, evidencing a decreased number of intraepidermal fibers in rodents and impaired corneal innervation in rats [43,138].

The positive sensory profile is associated to an increased number of ion channels, altered or impaired Ca^2+^ signalling, neuroinflammation and activation of adjacent silent nociceptors.

Within TIPN positive sensory symptom profile, neuropathic pain includes sensations of stabbing, burning, shooting, tingling, allodynia and hyperalgesia which are maintained by peripheral and central sensitization [26,139]. As taxanes are not able to cross the blood-brain barrier, in the first phases of pain, peripheral nociceptive inputs involve spinal cord and central nervous system; when taxanes start mitochondrial damage, abnormal spontaneous discharge of Aδ and C-fibers with mechano and thermal hypersensitivity via TRPA1 (mechano and cold sensitivity) and TRPV1 (heat hyperalgesia) receptors at the distal terminal sensory ending takes place [37], leading to a constant dorsal horn excitability (glia cell activation, microglia and astrocytes) [44], which seem to be central in maintaining neuropathic pain, increasing proinflammatory cytokines and brain-derived neurotrophic factor (BDNF) [45]. All these events result in cortical changes and central sensitization [140]. The inhibition of macrophages and microglia prevents the development of mechanical hyperalgesia and epidermal nerve fiber loss [44,46].

TIPN has surely different onset causes, whose role is differently weighted in different patients and clinical conditions. Microtubule impairment, neuroimmune and inflammatory changes, ion channel remodelling, impaired mitochondrial function and genetic predisposition are most of the likely involved mechanisms, also studied through animal and human research models.

Referring to taxane anticancer mechanisms of action, healthy peripheral nerves undergo a similar attack which compromises the dynamic stability of microtubules, a fundamental property for the anterograde transport of nutrients, neurotransmitters and mitochondria from nervous soma to axon extremities and for retrograde transport of used/empty synaptic vesicles and proteins from axons to soma. TIPN is associated to the loss of microtubule dynamic stability which leads to a loss of transport functions and translates into altered growth cone and filipodia at the sensory endings.

These alterations bring to changes in the morphology and physiology of the peripheral nerves which result in a symmetrical development in a stocking/glove distribution [9]. Longer nerves seem to be more vulnerable to impaired transport, in fact, distal axonopathy and *coasting* phenomenon are more frequent. The term *coasting* describes progressive worsening of TIPN symptoms, related to the time for anterograde transport to move the drug from the dorsal root ganglion (DRG) to the distal axon [32].

Gornstein and Swartz [16,24] suggest that transport block may not be the primary cause of paclitaxel-induced PN, based on the evidence that drug inclusion both in the middle and the distal axon in adult mouse DRG neurons scattered a higher sensitivity for the distal portion, while for the middle one no difference was evidenced with respect to control. The results of this study suggest that paclitaxel disrupts growth cone dynamics in filipodial extension of pioneer microtubules in the sensory nerve, leading to their stabilization and impairment at the distal axon. In normal conditions, pioneer microtubules are always in a high metabolic state, granting retraction and advancement of the nerve as the epithelium regenerates [27].

Other studies have suggested that primary mechanisms of TIPN are due to a second low-affinity binding site of taxanes at the end of the microtubules, whose activation scatters the release of mediators of inflammation (IL, NO, and COX-2) [47]. In particular, paclitaxel increases the production/release of pro-inflammatory cytokines (TNF alfa and IL-1 beta) and decreases anti-inflammatory cytokines (IL-4 and IL-10) [141,142]. As a consequence, immune cells activation and neuroinflammation take place [8].

This process includes mitotoxicity, in particular referring to the presence of swollen and vacuolated mitochondria in Aδ and C fibers which stimulates Ca^2+^ release, opening mitochondrial permeability transition pore (mPTP) and leading to mitochondria depolarization [48,143] and neuronal excitability [144]. This damage of axon functions induces a reactive oxidative stress, ion channel changes (TRPV1 via PKC or MAPK), release of inflammatory cytokines and activation of caspase 3/7 [18,19,43].

Damaged mitochondria start an oxidative stress with the production of reactive oxygen species (ROS), both in sensory neurons and the spinal cord [49,50,141,142], which activate apoptotic processes and promote the disruption of cell structure (and consequently demyelination), by a self-amplifying mechanism with further mitochondrial damage. Different studies demonstrated paclitaxel-induced swelling, vacuolation and loss of structure of mitochondria [37,51,52].

As for ion channel alterations, different models have evidenced a decreased expression of K^+^ channels which causes the spontaneous activity of nociceptors and the activation of cation channels TRPV1 and TRPA1, mediators of pain signalling, in DRG neurons [52,53,54]. To support these observations, antagonists of TRPA1 have demonstrated to reduce inflammation, cold allodynia and hyperalgesia [55,56,145].

In summary, TIPN is mediated by different drug-related effects:

- mitochondrial dysfunction
- axon degeneration
- altered Calcium homeostasis
- changes in peripheral nerve excitability
- immune processes and neuroinflammation

Nerve conduction studies, which measure amplitude and velocity of large myelinated fibers, are not sufficient to monitor changes in sensory neuropathies [23,146], in fact, the early stages of the axonopathy and the worsening clinical conditions over time can be better highlighted through a skin biopsy, quantification of intraepidermal nerve fiber density (IENFD) and quantitative sensory testing (QST). In particular, presence of symptoms with abnormal QST and a normal nerve conduction study are generally sufficient to recognize the diagnosis of small-fiber neuropathy [23].

Antioxidants, vitamins A, B, and E, herbal medicines such as Goshajinkigan, acetyl-l-carnitine, Ca^2+^-Mg^2+^ infusions, acupuncture, have been tested for the treatment of TIPN [15,147], but they failed to produce either neuroprotection or alleviation of symptoms [57,148]. Krukowski [148] showed that IL-10 can attenuate paclitaxel-induced CIPN. Practice guidelines from the American Society for Clinical Oncology in 2014 and a consensus statement from the Canadian Pain Society have suggested moderate treatment support with duloxetine, a serotonin-norepinephrine reuptake inhibitor [149,150,151] in association to physical therapy programs and psychological treatment [152], while no kind of prevention is recognized.

Topical drugs have been also tested to treat TIPN sensory symptoms, with contrastant results from clinical trials:

(a) topical ketamine and amitriptyline demonstrated no improvement in pain scores [153];
(b) topical combination of ketamine, amitriptyline and baclofen demonstrated improved tingling sensory subscales, but no improvement in numbness, thermal pain or functional abilities [154];
(c) topical menthol has been suggested but requires further study [155].

The reason why no ancillary treatment is still recognized lies on the general consideration that targeting the pathway to prevent or minimize TIPN can also affect taxane drug efficacy.

A possible neuroprotective role of physical exercise in TIPN has been reported. A risk analysis reported by Mustafa [156] in breast cancer patients has revealed a 12% lower risk of peripheral neuropathy for women who regularly made physical exercising of at least 30 min. Identifying specific protocols of physical exercise intensity, frequency, duration and type is one of the best routes for prevention and treatment of TIPN and neuropathic pain, above all for patients’ compliance.

### 4.2. Platinum Salts

Cisplatin, carboplatin and oxaliplatin are among the most prescribed chemotherapic drugs for the treatment both of solid and hematologic tumors; in particular, cisplatin and carboplatin are used for small-cell/no-small cell lung, testicular, ovarian, uterine and bladder cancers, but also in refractory lymphoma in DHAP schedule, while oxaliplatin in digestive tract tumors, such as colorectal, oesophagal, stomach, liver, pancreatic cancers and in refractory lymphoma in DHAOX schedule. Cisplatin-induced peripheral neuropathy (CisIPN) is time-, dose and patient-dependent with symptoms appearing after the first dose or after 12 cycles of therapy [60,157], independently from pretreatment, age, sex, tumor type and cotreatment with other chemotherapeutics [61,157]. It is generally recognized that more severe forms of CisIPN appear after cumulative doses above 350 mg/m^2^ with the most frequency in 92% of patients at a cumulative dose of 500–600 mg/m^2^ [62]; a form of chronic CisIPN has been also observed in 5–20% of patients at 12 months after therapy [63,157]. Carboplatin develops a less severe neuropathy observed in 13–42% of the treated patients [158], while oxaliplatin induces an acute and transient OIPN (oxaliplatin-induced peripheral neuropathy) in 65–98% of patients in the following hours after each infusion at a dose range from 85 to 130 mg/m^2^ and lasting up to 5–7 days, with a longer persistence of symptoms in patients receiving 12 cycles of chemotherapy [64,65]. Acute OIPN presents with cold-related paresthesias of the extremities (hands and feet), pharyngolaryngeal dysesthesias, jaw spasms, fasciculations and muscle cramps [159]. Cold-induced neuropathy represents the most important difference between oxaliplatin and cisplatin-induced neuropathy [66] and its severity in the first phases of exposition has been associated to the chronic form of OIPN experienced one year later [67], observed in 50–70% of patients, depending on the intensity of symptoms assessed in its acute clinical presentation [68,69,70]. A systematic review by Beijers et al. [71], in addition to the study by Briani et al. has reported that OIPN may be present in 26–46% of patients at the 12-month follow-up, in 24% of patients at the 15–18-month follow-up and even in 84% of patients at the 24-month follow-up [71,72,73].

Chronic OIPN occurs in 10% of patients who received cumulated doses from 510 to 765 mg/m^2^, increasing to almost 50% of patients for more than 1000 mg/m^2^ doses [74,75] and specifically a more severe acute form results in a higher incidence of chronic neuropathy [75].

Gene alterations, such as GSTP1 and GSTM1, glutathione-S-transferase genes and voltage-gated sodium channel genes SCN4A, SCN9A and SCN10A, together with pre-existing peripheral neuropathy [75,76,77,78] have been demonstrated to be the principal risk factors for OIPN onset.

As for pathogenetic mechanisms for CIPN, like taxanes, platinum salts induce the activation of glial cells, which on turn provoke the activation of immune cells and the release of pro-inflammatory cytokines (interleukins and chemokines), leading to the nociceptor sensitization and hyperexcitability of peripheral neurons, together with blood-brain barrier damage, also mediated byROS, till the development of neuroinflammation. Platinum salts also start a mitochondrial damage with the disruption of the respiratory chain function and an increased production of ROS.

In detail, oxaliplatin and cisplatin form abnormal adducts binding to mitochondrial DNA (mDNA) both in neuronal and non-neuronal cells, which cannot be repaired as mitochondria have no DNA repair system. They start transcription of mDNA, leading to abnormal proteins, impairment of the respiratory chain in mitochondria [79,80,81,82,160,161], an increased production of ROS (superoxide anion in particular) and oxidative stress [58,83,84,162], lipid peroxidation, protein and DNA oxidation in both sciatic nerves and the spinal cord. ROS can also accompany a dysregulation of calcium homeostasis and signalling pathways, inducing apoptotic changes in peripheral nerves and in DRG cells. An increase of intracellular Ca^2+^ concentration may result in calpain (a potent protease) activation, which leads to unregulated proteolysis, directly triggering axon degeneration [163]. Moreover, oxalate deriving from oxaliplatin hydrolysis is a Ca^2+^ chelator and may lead to an increase in Na+ conductance. Calcium homeostasis alterations also modify the function of protein kinase families (MAPK, mitogen-activated protein kinases; JNK, c-Jun N-terminal kinase; PKC, protein kinase C; AKT, serine-threonine kinases). Platinum-based drugs also modify Na^+^, K^+^ and TRP ion channel activity, inducing the hyperexcitability of peripheral neurons and axonal degeneration [85,86].

These toxic effects are part of the mechanisms of action of platinum drugs as anticancer drugs, so that are almost inevitable [87,88].

In the case of oxaliplatin, animal models also demonstrated that the rapid non-enzymatic detachment of the leaving-group oxalate, leading to reactive platinum complexes, has an important role in neuropathy onset, contributing to the characteristic acute cold-induced neuropathy [88]. The results of Fujita et al. also indicate that cells overexpressing Octn1 and Mate1, the recognized oxaliplatin transporters, bring to platinum accumulation in DRG neurons, followed by OIPN. The accumulation of platinum is proportionally correlated with the severity of neuropathy in laboratory animals [89].

IL-1b, IL-6 and TNF-a levels, produced by activated glial cells are able to start neuropathic pain [90,164,165,166] thanks to platinum salts’ ability to activate the Toll-like receptor (TLR) family, especially TLR4. In knockout mice lacking that receptor, pain induced by cisplatin was decreased [91]. Jin’s study confirmed that pro-inflammatory cytokines sensitize nociceptors by the modulation of ion channel propertie [167]. Moreover, these cytokines can arrive also to spinal and supraspinal levels [92] chemokine ligands are responsible for the migration and infiltration of monocytes/macrophages and other immune cells, thus contributing to neuroinflammation and pain behaviour in animal models. Other studies have confirmed the role of chemokine CXCL12 [93] and chemokine CX3CL1 signalling in OIPN [94].

Recently, a new neuropsychological syndrome/cognitive impairment has been associated to cancer treatment with platinum salts [95,168,169,170,171]. Oxaliplatin theoretically does not pass through blood-brain barrier (BBB), but damage may be caused by proinflammatory cytokines, ROS or other neurotransmitters [172,173]. Branca et al. [96]. demonstrated that oxaliplatin administration in vitro significantly altered junctional and cytoskeletal apparatus of endothelial cells, leading to a higher drug concentration in the brain, which probably contribute to pain chronification. The study of Sanna et al. shows that oxaliplatin administration induces changes in the levels of proteins in spinal and supraspinal levels in laboratory animals and suggests a direct correlation between structural changes in the central nervous system and chemotherapy-induced neurotoxicity [97].

A systematic review by Hou describes 26 different treatment options for cisplatin-induced IPN, including pharmacological, light, scrambler, magnetic field, acupuncture, dietary and long-wave diathermy therapy [174]. However, duloxetine is the only one drug recommended by the American Society of Clinical Oncology, while photobiomodulation or low-level laser therapy is only occasionally prescribed for treatment of CIPN. Apart these options, a lengthy list of other treatments showed no appreciable value to CIPN sufferers (topical 4% ketamine and 2% amitriptyline, tricyclic antidepressants, andamitriptyline, and nortriptyline) [174], besides many antioxidants, such as vitamin B6, vitamin E, omega-3 fatty acids, glutamine, glutathione, acetyl-L-carnitine and α-lipoic acid [175,176].

Amifostine was also tested for prevention of CIPN, because one of its active metabolites, WR1065, has been demonstrated to protect differentiated neurons from cisplatin-induced damage [98]. This thiol accumulates in normal tissues more than in tumors, protecting them from side effects of radiation and chemotherapy without decreasing their therapeutic efficacy [177].

Finally, among novel treatments for CIPN, manganese chelates are noted to be mimetics for mitochondrial manganese superoxide dismutase and efficient in ROS reduction [99,178].should discuss the results and how they can be interpreted in perspective of previous studies and of the working hypotheses. The findings and their implications should be discussed in the broadest context possible. Future research directions may also be highlighted.

### 4.3. Bortezomib

Bortezomib is an N-protected dipeptide containing a boronic acid instead of a carboxylic acid and is an anti-cancer drug for the treatment of multiple myeloma and mantle cell lymphoma as first and further line treatment, often co-administered within a complex medical protocol together with other medications [100,101]. Together with carfilzomib, bortezomib is a reversible proteasome inhibitor thank to its boron atom which binds the catalytic site of the26S proteasome [100,102].with high affinity and specificity, blocking the degradation of cellular proteins and ridding cells of abnormal or misfolded proteins.

Both subcutaneous and intravenous injection provide equal drug exposures and generally comparable therapeutic efficacy administration, though in the former peak plasma levels are ~25–50 nM for 1–2h and in the latter case peak plasma levels are ~500 nM for only ~5 min, after which the drug rapidly distributes to tissues with a large volume of distribution (~500 L) [101,102] and an elimination half-life of 9–15 h. In the light of these data, subcutaneous formulation evokes a lower incidence of neuropathy [103,104]. Retrospective analyses have been made to deepen in the possible differences in neurotoxicity between the two routes [105,106,107]: clinical data are not uniform, in some cases [105] no differences have been evidenced, in others [106,107] a high or higher important incidence of PN has been reported for intravenous with respect to subcutaneous route.

Bortezomib-induced PN generally starts early, often within 3–4 cycles of treatment with a peak around cycle 5, followed by a plateau, but also resolves or improves in two-thirds to three-quarters of the patients after a median of 2 to 3 months. This trend suggests a neurotoxic dose threshold rather than a cumulative dose effect and a dose adjustment may be necessary with increasing toxicity.

PN frequency is approximately 34% of treated patients, who develop chronic, distal and symmetrical sensory syndrome with an important neuropathic pain that may last even for years after drug administration [108]. Also a length-dependent, mixed small and large fiber axonal sensory neuropathy can take place and disappear after several weeks after treatment ends. SUMMIT and CREST clinical trials are the most reliable reports on the outcome of bortezomib-induced PN on 256 patients. Within the study population, 90 patients experienced treatment-emergent peripheral neuropathy, in particular, 35 experienced either grade 3 or 4 neuropathy and/or neuropathy leading to discontinuation of treatment (5%, 14/256), while dose reduction was required in 12% of patients (31/256). In 7% of patients (19/256), at least one course of bortezomib was omitted because of peripheral neuropathy.

Other mechanisms of neurotoxicity have been proposed to clarify bortezomib-induced PN, one of which regards its ability to increase sphingolipid metabolism within astrocytes with a subsequent increasing levels of ceramide, sphingosine-1 phosphate (S1P) and dihydrosphingosine-1-phosphate (DH-S1P), that have potent inflammatory and nociceptive actions [109]. S1P receptor-1 (S1PR1) signalling pathways contribute to bortezomib-induced neuropathic pain, because when it hooks to free S1P, an increase in the release of presynaptic glutamate at the level of the dorsal horn of the spinal cord takes place, contributing to the development of neuropathic pain [109]. Alterations in sphingolipid metabolism is often associated to neuronal degeneration and pain, for instance, the mutations in serine palmitoyltransferase (SPT) gene that notoriously lead neuropathic pain in humans [179].In the periphery, bortezomib is able to augment tumor necrosis factor α (TNF-α) and interleukin-1β production, which in turn have an important role in sphingolipid metabolism. Mitochondrial damage also resulted from bortezomib treatment, giving ROS, which in turn, impair mitochondrial function, like the prior discussed taxanes and platinum salts. Thanks to its cytotoxic mechanism of action, bortezomib also activates the mitochondrial-based apoptotic pathway, dysregulates mitochondrial-mediated Ca^++^ homeostasis, provokes chromatolysis of DRG neurons, followed by cytoplasmic accumulation of eosinophilic material, neurofilaments and juxtanuclear electron-dense cytoplasmic deposits [110].

Some SNPs and other clinical conditions have been identified to clarify the severity and the late *versus* early-onset bortezomib neuropathy, demonstrating the probable role of pre-existing genetic factors [111]:

- a locus in the PKNOX1 gene and one near the CBS gene [112];
- the inhibition of the nuclear transcription factor NFκB, involved in cell survival and proliferation [113];
- low levels of vitamin D, which are prognostic for a more severe neuropathy in myeloma patients [114].

To date, dose reduction and schedule change algorithm with weekly administration rather than 1–4-8–11 days within a cycle, are the only clinical approaches to face bortezomib-induced PN, as neither neuroprotective agents demonstrated their efficacy nor analgesics, tricyclic antidepressants, anticonvulsants or vitamin supplements as symptomatic treatments gave sufficient results.

There are recommendations concerning the use of nutritional supplements, such as pyridostigmine (vitamin B6) and vitamin C that should be omitted in the course of treatment: vitamin B6 can cause additional sensory neuropathy in patients with impaired renal function and in association with a protein-deficient diet, while vitamin C may interfere with bortezomib metabolism and its proteasome activity.

Interestingly, also intravenous immunoglobulin (IVIG) administration has been proposed by a preliminary report on 9 patients as an effective therapy for symptomatic management of bortezomib-induced PN, being evident an improvement of at least 1 grade in all patients.

### 4.4. Thalidomide

Thalidomide is a glutamic acid derivative for the treatment of multiple myeloma [117], thanks to its immunomodulatory activity, which blocks both the production of tumor necrosis factor-alpha (TNF-α) and the activation of NF-kB (nuclear factor kappaB), resulting in the dysregulation of neurotrophins with their receptors which accelerates neuronal cell death [180]. Thalidomide has also an antiangiogenic activity by the inhibition of basic fibroblast growth factor (b-FGF) and vascular endothelial growth factor (VEGF), causing secondary ischemia and hypoxia of nerve fibres and, subsequently, irreversible damage of sensory neurons [181].

When Wani [118] proposed the role of the dihydroxy metabolite of thalidomide in extensive redox-activated DNA cleavage through the formation of ROS as probable guilt for the well-known thalidomide-induced teratogenesis, a series of preclinical and clinical trials have started to study ROS-dependent mechanisms also for the onset of PN, being typical of the most neurotoxic chemotherapeutics.

Thalidomide-induced peripheral neuropathy occurs in 25–75% of patients, with dose-dependent prevalence and severity [182], in particular in advanced-aged patients with pre-existing neuropathy, responsible for a limited treatment duration [119]. More than other drugs, thalidomide also induces motor impairment and gastrointestinal and cardiovascular autonomic manifestations [182]; all these effects lead approximately 15% of patients to treatment discontinuation [120]. The median time to onset is usually months, but higher doses accelerate the process, especially in patients with relapsed disease, whose incidence doubles after 6 to 12 months of thalidomide exposure. Recovering from this nerve damage is variable, taking 4 to 6 years for only a quarter of patients and up to 3 years for the first signs of improvement.

Some genetic studies have evidenced that ADME gene group (drug Absorption, Distribution, Metabolism and Excretion), cytochromes and genes involved in neural processes and central nervous system development can possible risk factors for the onset and progressingof thalidomide-induced peripheral neuropathy [115], as well as single nucleotide polymorphisms (SNPs) in genes involved in inflammation and repair mechanisms in the peripheral nervous system [121].

The association bortezomib and thalidomide is a common therapeutic strategy in multiple myeloma [104,114,115]. Though their common activity in peripheral neuropathy induction, some studies have evidenced that no incremental effects are experienced by patients [116]. Finally, a retrospective study suggested a lower incidence of PN outcomes and superior efficacy with subcutaneous bortezomib combined with thalidomide and dexamethasone (VTD) in MM when compared with intravenous route [115,116,122].

### 4.5. Epothilones

Epothilones, in particular ixabepilone, are tubulin destabilizers like taxanes, binding preferentially to the beta III tubulin isotype. In the United States, FDA approved ixabepilone for the treatment of breast cancer not responding to other available chemotherapies [14,147], while in Europe this class of drugs didn’t received any approved clinical application. The prevalence of CIPN for ixabepilone has been estimated approximately 67%, with an incidence of severe CIPN from 1% in previously untreated patients to 24% in pretreated patients [14]. The clinical manifestation of CIPN is similar to taxane, sharing similar mechanism of action both in anticancer and in neuronal damage activity [14]: in particular mild to moderate sensory dominant neuropathy, mostly affecting small-diameter sensory fibers, in form of paresthesias, numbness and pain in the feet and hands (stocking-and-glove distribution), while motor involvement is rare, manifesting as mild muscle weakness and autonomic manifestation was observed in less than 1% of patients. These symptoms are dose-dependent and are reversible with treatment discontinuation [14]. Intuitively, the pathomechanisms for PN induced by epothilones are also similar to taxanes:

- microtubule disruption impairs axonal transport and leads to Wallerian degeneration;
- altered activity of ion channels and hyperexcitability of peripheral neurons;
- mitochondrial damage, which increases the production of ROS that insult neuronal enzymes, proteins and lipids as well as provoke apoptotic changes and altered excitability to peripheral nerves [183];-ROS release stimulates the attraction and activation of T-lymphocytes and monocytes, as well as pro-inflammatory cytokines (interleukins and chemokines), which are responsible for neuroinflammation.

Being epothilone-induced PN severity milder than taxanes [122], it is generally accepted that off-target mechanisms responsible for taxane-induced neuropathy may not be shared for epothilones, but the question is still under investigation.

### 4.6. Vinca Alkaloids

Vincristine, vinblastine, vinorelbine and vindesine are the well-known phase M cycle-specific drugs belonging to this class of chemotherapeutics, commonly prescribed in the treatment of different tumors, such as Hodgkin and non–Hodgkin lymphoma, testicular cancer and non–small cell lung cancer. In analogy to taxanes, vinca alkaloids bind with tubuline, but blocking its polymerization and assembly into microtubules cell and lead to disrupting axonal transport. Though unable to readily cross the blood-brain barrier, they can act on the soma of peripheral nerves, generating a dose-dependent sensorimotor neuropathy, with symptoms’ onset (usually pain in hands and feet) within the first months of treatment. Patients also experienced muscle weakness, including wrist extensors, dorsiflexor weakness and cramping [8].

Vincristine is generally considered the most neurotoxic drug among vinca alkaloids, followed by vinblastine and vinorelbine: its neurotoxic effects involve both sensory (early symptoms at cumulative doses of 4 mg/m^2^) and motor (distal weakness after 6–8 mg/m^2^) and autonomic fibers in up to one-third of patients [123]. Vincristine-induced neuropathic pain is principally due to axonal distal neuropathy by disrupting the microtubular both fast and slow axonal transport system, but also a reduction of sensory nerve action potential amplitude has been reported [124]. Vincristine also induces ultrastructural changes in the cytoskeleton of large myelinated axons and an accumulation of neurofilaments in dorsal sensory ganglion neurons [125,184]. Both sensory and motor neuropathy with vinorelbine is usually milder than vincristine and has been reported in 14% to 48% of patients receiving a total dose of 30 mg/m^2^ [126].

The recognized and principal pathogenetic mechanisms of CIPN by vinca alkaloids are:

- changes to large axons and DRG neurons, which leads to Wallerian degeneration;
- altered activity of ion channels and hyperexcitability of peripheral neurons;
- inhibition of polymerization into microtubules inhibits axonal transport, generating distal
- axonopathy and altered excitability;
- attraction and activation of immune cells start the release of pro-inflammatory cytokines (interleukins and chemokines), inducing neuroinflammation.

FDA issued a black box warning on vincristine prescription in Charcot–Marie–Tooth disease (CMT) patients [127], having the ERG2 gene mutation, who have been demonstrated more sensitive to vincristine [59], as well as a polymorphism in the CEP72 gene, which is associated with an increased risk and severity of vincristine-induced neuropathy [128].Due to their hepatic metabolism by CYP3A4 enzymes, a concomitant use of taxanes or CYP3A4 inhibitors (Ketoconazole, clarithromycin, ritonavir) increases the risk of vinca alkaloids-induced neuropathy, together with a decreased serum level of folic acid and vitamin B12, pre-existing neuropathy and comorbidities, such as diabetes, smoking history and low creatinine clearance. There is no specific treatment for vinca alkaloid induced peripheral neuropathy. The use of pyridoxine or pyridostigmine revealed a certain efficacy in vincristine-induced neuropathy [129]. A topical cream containing capsaicin demonstrated to give benefit in PN [130,185], thanks to the depletion of P substance in the distal nerve endings. In the case of paraesthesia and pain, the patient should be treated with carbamazepine, imipramine or lignocaine [186].

### 4.7. Tyrosine Kinase Inhibitors: Sorafenib

A tyrosine kinase inhibitor (TKI) is an anticancer drug able to inhibit tyrosine kinase receptors, which are endowed of an enzymatic activity, responsible for the activation by phosphorylation of many proteins by signal transduction cascades. TKIs operate by different mechanisms:

- competition withthe physiological ligand adenosine triphosphate(ATP) on the active site within the receptor;
- binding outside the active site (allosteric site) of the enzymatic portion of the receptor, affecting its activity and conformational status [187];
- depriving tyrosine kinases of access to the Cdc37-Hsp90 molecular chaperone system on which they depend for their cellular stability, leading to their ubiquitylation and degradation [188].

With an incidence of >10%, several oral TKIs can induced PN: brigatinib, crizotinib, encorafenib, imatinib, ivosidenib, ixazomib, lorlatinib, ponatinib, vemurafenib, sorafenib [189,190,191,192,193,194]. Sorafenib is a TKI anticancer drug approved in clinics for the treatment of hepatocellular and renal carcinomas, whose repeated administration causes the onset of a peripheral painful neuropathy, which often leads to premature discontinuation of therapy, being resistant to common analgesic drugs [131,195]. The pathomechanism for this PN is still under investigation, but the first studies have identified a direct neurotrophic role for vascular endothelial growth factor (VEGF). VEGFR inhibition by sorafenib seems to be responsible for peripheral sensory neuropathies, interfering with the endogenous neuroprotective action of VEGF on sensory neurons. This role of VEGF/VEGFR interaction provides an unprecedented evidence that VEGF receptor inhibitors may themselves trigger a painful neuropathy and aggravate paclitaxel-induced neuropathies, when used in combination. For the same reason, also the anti-VEGF bevacizumab in association to paclitaxel precipitates taxane-induced PN [132,196]. The disruption of the neuroprotective effect of VEGF leads to neuronal stress and apoptosis through a mechanism involving VEGF receptor-2-mediated expression of the anti-apoptotic protein Bcl2 [197]. Due to this peculiar pathogenetic mechanism which is also its biological target as anticancer drug, sorafenib-induced PN can be modulated only with dose reduction and often resolved by treatment discontinuation.

All the described drugs or class of drugs induce CIPN preferentially in patients with comorbilities. Neither drugs in therapy or prevention have succeeded in ameliorating sharply neuropathic symptoms and pain, nor quantitative assessment of neuropathy has been authorized or approved so far. Nevertheless, since taxanes and platinum salts are known to hit first small autonomic C-fibers, some efforts have been made to quantify neuropathic damagein the early steps of the anticancer treatment [133,198,199,200]. Recently, a sudomotor function testing has been suggested to screen early peripheral neuropathy, on the basis that sweat glands are innervated by small C-fibers,. Other assessment tools have already been reported in literature [198,199,200]:

- skin biopsy to determine the intradermal nerve fiber density and innervated skin structure;
- corneal confocal microscopy to quantify small nerve fibers in the cornea;
- sympathetic skin response, measured the unmyelinated nerve sudormotor function after a transient change in electrical skin potential;
- axon flare reflex tests, evidencing the hyperemic response of the axons;
- pain-related evoked potentials to assess the nociceptive system.

If the route to quantify neurotoxicity induced by these drugs has recently brought to some results, the treatment of CIPN is still far from a common consensus.

This paper is a starting point for opening in our hospital a new scenario for the preventive evaluation of neuropathic onset and has energized our efforts to start creating a database with real data about our patients. In the next future, these data together with clinical outcomes will be object of an original publication.

## 5. Conclusions

Chemotherapy-induced painful neuropathy is a major side effect of several chemotherapeutic agents commonly administered by most patients. To prevent or alleviate its symptoms without limiting the potentially life-saving therapy is a due aim for oncologists, physicians and hospital pharmacists who prescribe and manage anticancer drugs. The negative impact on patients’ quality of life constitutes a major problem both for the path of cure they are passing through and for their health care providers. For this reason, in high-risk patients, a neurological examination with electrophysiological evaluation should be done early and as soon as first symptoms appear.

Different drugs have been tested to reduce the incidence or severity of CIPN, but none of them are currently applied or strongly recommended. In the ASCO guidelines, no agent for the prevention and only a moderate recommendation for duloxetine in treatment are present.

Understanding CIPN pathomechanisms and risk factors is the only route to identify the most susceptible patients and stimulate research in new possible treatments, together with next advances in genome-wide association studies (GWAS), that could permit the identification of critical genes involved in biological pathways leading to peripheral nerve damage [201]. In the future, a translation of the GWAS results would allow the prediction of CIPN occurrence during chemotherapy administration and could address also to target therapies for managing it.

## Figures and Tables

**Table 1:** Principal anticancer drugs inducing PN with their pathologic and pharmacologic characteristics.

Class of Drugs	Mechanism of Action	Application in Oncology	Mechanism CIPN Onset	Prevalence of CIPN	Risk Factors	References
**Taxanes** (**paclitaxel docetaxel**)	Antimitotic effect due to tubulin binding (microtubule stabilizer)	breast, ovarian, uterus, gastric cancers, NSCLC, sarcomas,	Microtubule impairment, loss of axon transport function, neuroimmunity, inflammation (NO,IL-1,TNFa,COX2), ROS production, ion channel remodeling, Ca++ signaling, mitochondrial dysfunction	chronic sensorial	Genetic predisposition, advanced age, smoke, pre-existing neuropathy diabete, autoimmunity	[16,22,24,25,31,32,33,34,35,36,37,38,39,40,41,42,43,44,45,46,47,48,49,50,51,52,53,54,55,56,57,58,59]
**Platinum salts** (**oxaliplatin and cisplatin**)	Antimitotic effect due to alkylation of DNA N7 Guanine	colon, pancreas, gastric, pulmonary, ovarian,	neuroimmunity, inflammation (NO, IL-1, TNFa, COX2), ROS production, ion channel remodeling, Ca++ signaling, mitochondrial dysfunction, protease activation, altered brain endothelial cells	Acute/chronic motor, sensorial	Genetic alterations on GSTP1, GSTM1, glutathione-S- transferase, Na+voltage channels, pre-existing neuropathy	[41,43,51,58,60,61,62,63,64,65,66,67,68,69,70,71,72,73,74,75,76,77,78,79,80,81,82,83,84,85,86,87,88,89,90,91,92,93,94,95,96,97,98,99]
**Bortezomib**	Proteasome 26S inhibitor	multiple myeloma, lymphomas	Increased sphingolipid metabolism in astrocytes, inflammation (TNFa, IL-1), mitochondrial damage, ROS production, alteration in Ca++ signaling	Chronic sensorial	SPT, PKNOX1 and CBS gene alterations, low levels of vitamin D, inhibition of NFkB	[41,100,101,102,103,104,105,106,107,108,109,110,111,112,113,114,115,116]
**Thalidomide**	Immunomodulator effect	multiple myeloma,	ROS production	Acute/chronic; motor and autonomic	Advanced age, pre-existing neuropathy, ADME gene group alteration, SNPs	[100,104,114,115,116,117,118,119,120,121]
**Epothilones** (**ixabepilone**)	Antimitotic effect due to tubulin binding (microtubule stabilizer)	breast cancer	See Taxanes	Acute sensorial	See Taxanes	[14,122,123]
**Vinca Alkaloyds** (**vincristine, vinorelbine, vinblastine**)	Antimitotic effect due to tubulin binding (preventing microtubule polymerization)	lymphomas, breast cancer	Microtubule impairment, loss of axon transport function, altered ion channels, neuroinflammation	Acute sensorial motor and autonomic	ERG2 gene mutation, polymorphism in CEP72 gene, diabete, smoke, decreased serum level of folic acid and vitamin B12, pre-existing neuropathy, low creatinne clearance	[41,42,111,124,125,126,127,128,129,130]
**TKIs** (**Sorafenib**)	Kinase inhibitor (proliferation and angiogenesis inhibition)	liver and kidney cancers	VEGFR inhibition, expression of Bcl2	Acute	n.a.	[131,132,133]

## Data Availability

Not applicable.
